# Hierarchical-circular model of biological memory: a multilevel hypothesis for pathogenesis and allostatic integrity in Alzheimer’s disease and related dementias

**DOI:** 10.3389/frdem.2026.1841647

**Published:** 2026-06-24

**Authors:** Samuel Ruesga Mundo

**Affiliations:** High Specialty Medical Unit, Western National Medical Center, Mexican Institute of Social Security (IMSS), Guadalajara, Jalisco, Mexico

**Keywords:** Alzheimer’s disease, dementia, allostatic load, epigenetic memory, interoception, biological memory, multilevel model, PNEI network

## Abstract

**Introduction:**

Alzheimer’s disease and related dementias remain largely resistant to disease-modifying therapies, despite decades of research focused on linear neuropathological pathways such as beta-amyloid and tau. Persistent paradoxes—including the dissociation between pathology burden and clinical expression, the impact of early-life stress, and the role of systemic factors—indicate the need for integrative theoretical frameworks. This article proposes a multilevel hypothesis conceptualizing dementias as disorders of biological memory and allostatic integrity rather than isolated brain pathologies.

**Hypothesis:**

The Hierarchical-Circular Model of Biological Memory posits that dementia emerges from progressive disruptions in a circular, multilevel system that encodes and stabilizes biological information across the lifespan. The model is organized around the unifying principle “Signal → Plasticity → Stable State” and integrates five interconnected levels: (1) morphogenetic programming and genetic architecture, (2) epigenetic molecular memory, (3) allostatic load and systemic physiological adaptation, (4) the Psychological–Neurological–Endocrine–Immunological (PNEI) network, and (5) interoceptive–neuronal integration. At any level, perturbation can propagate bidirectionally through the system, establishing maladaptive stable states that manifest clinically as dementia.

**Development of the hypothesis:**

Through a structured synthesis of longitudinal, mechanistic, and multisystem studies (2010–2025), the model specifies how gene–environment interactions, epigenetic modifications, cumulative allostatic load, neuroimmune dynamics, and altered interoceptive timescales jointly shape vulnerability and resilience. The concept of allostatic integrity is introduced as a dynamic systems-level property—distinct from allostatic load—that explains why similar neuropathological burdens may result in divergent clinical trajectories. Distinct dementia phenotypes are proposed to reflect different patterns of circular reinforcement across the five levels.

**Testable predictions:**

This framework generates concrete, falsifiable predictions: (1) composite indices of allostatic integrity will outperform single biomarkers in predicting conversion from mild cognitive impairment to dementia; (2) multidomain interventions targeting more than one system level will have multiplicative, rather than additive, effects on slowing cognitive decline; (3) patients with similar amyloid/tau profiles but contrasting allostatic integrity will show markedly different trajectories of clinical progression; and (4) allostatic integrity moderates the protective effect of cognitive reserve, a pattern not predicted by reserve frameworks alone.

**Conclusion:**

The Hierarchical-Circular Model of Biological Memory offers a unifying hypothesis for Alzheimer’s disease and related dementias that bridges genetic, epigenetic, physiological, neuroimmune, and interoceptive processes across the lifespan. By reframing dementias as failures of biological memory and allostatic integrity, the model provides a conceptual roadmap for mechanistic research, multidomain prevention, and personalized treatment strategies.

## 1 Introduction

Alzheimer’s disease and related dementias (ADRD) represent one of the greatest public health challenges of the 21st century, with global prevalence expected to rise sharply in the coming decades (GBD 2019 Dementia Forecasting Collaborators, 2022). Despite unprecedented advances in neuroimaging, biomarkers, and genetics, disease-modifying therapies have produced only modest clinical benefits, and most pharmacological trials continue to fail (Cummings et al., 2024). This therapeutic impasse has fueled growing recognition that predominant linear models—centered on beta-amyloid, tau, and single-pathway cascades—are insufficient to capture the full complexity of dementia pathogenesis (Long and Holtzman, 2019).

Recent conceptual and diagnostic advances have deepened, rather than resolved, this paradox. The 2024 National Institute on Aging–Alzheimer’s Association (NIA–AA) biological criteria formally define Alzheimer’s disease by its underlying pathology and biomarker profile, rather than by clinical symptoms (Jack et al., 2018; Jack Jr et al., 2024). This shift has clarified the biological continuum of disease but has also highlighted a persistent dissociation between neuropathological burden and clinical expression: individuals with substantial amyloid and tau pathology may remain cognitively intact, whereas others with relatively modest biomarker changes exhibit rapid decline (Savva et al., 2009). These discrepancies suggest that dementia cannot be fully understood as a simple downstream consequence of aggregated proteins, but instead reflects broader disturbances in how biological systems encode, integrate, and stabilize information over time.

Converging evidence points to the importance of lifespan, multisystem influences that are only partially addressed by current frameworks (Livingston et al., 2020; Fratiglioni et al., 2020). Large longitudinal cohorts now show that elevated allostatic load—a composite index of cumulative physiological “wear and tear” across metabolic, cardiovascular, and inflammatory systems—is prospectively associated with increased risk for all-cause dementia and specific subtypes, including Alzheimer’s disease and vascular dementia (Ruan et al., 2025; Smith et al., 2025; Pérez-Carrasco et al., 2025). These findings support the view that chronic stress physiology functions as a key mediator between environmental exposures and late-life cognitive decline (Souza-Talarico et al., 2025). At the same time, emerging studies link adverse experiences across the life-course, including childhood abuse and adult economic hardship, to higher dementia risk (Livingston et al., 2020; Lee et al., 2025), underscoring the role of social and psychological stressors in shaping long-term vulnerability.

At the molecular level, gene–environment interactions and epigenetic mechanisms provide a plausible route by which early and chronic stress exposures become biologically embedded (Nöthling et al., 2025; Yusupov et al., 2024). Work on stress-responsive genes such as FKBP5 and risk alleles like APOE-ε4 demonstrates that specific genetic variants modulate epigenetic plasticity, hypothalamic–pituitary–adrenal (HPA) axis reactivity, and neurophysiological activity patterns in aging populations (Zannas et al., 2016; Wang et al., 2022; Verduzco Espinoza et al., 2025). These data reinforce the idea that genetic architecture and epigenetic “memory” jointly calibrate stress responses and neural network function across the lifespan, thereby influencing who will convert from mild cognitive impairment to dementia under comparable neuropathological conditions (Panitch et al., 2024; Klengel et al., 2013).

In parallel, advances in systems neuroscience and interoception research suggest that dementias involve fundamental disruptions in how the brain tracks, predicts, and regulates bodily signals (Critchley and Garfinkel, 2017; Khalsa et al., 2018). Recent work in behavioral-variant frontotemporal dementia has shown altered intrinsic neural timescales of interoceptive processing, pointing to a breakdown in allostatic–interoceptive networks that support adaptive regulation of internal states (Hazelton et al., 2025a, b). Similar alterations in interoceptive accuracy and network connectivity have been implicated in Alzheimer’s disease (Hazelton et al., 2025a, b), suggesting that disturbances in the dynamic integration of bodily and environmental information may constitute a transdiagnostic mechanism of cognitive and behavioral decline.

Taken together, these strands of evidence reveal a fragmented but convergent picture: dementia risk and progression are shaped by interactions among genetic predisposition, epigenetic molecular memory, cumulative allostatic load, multisystem (psychoneuroendocrine–immune) dynamics, and interoceptive–neuronal integration (McEwen and Stellar, 1993; Danese and McEwen, 2012; Slavich and Irwin, 2014; Feng et al., 2025). However, current models typically examine these components in isolation, lacking a unifying framework capable of explaining how perturbations at one level propagate across others to produce stable, maladaptive states (Long and Holtzman, 2019; Serrano-Pozo et al., 2024). The field therefore lacks an integrative language to describe dementia not merely as neurodegeneration, but as a systems-level failure of biological memory and allostatic integrity—the capacity of the organism to maintain adaptive circular information flow across multiple levels of organization.

In this article, a Hierarchical-Circular Model of Biological Memory is proposed as a multilevel hypothesis for Alzheimer’s disease and related dementias. The model is organized around the unifying principle “Signal → Plasticity → Stable State” and articulates five interconnected levels: (1) morphogenetic programming and genetic architecture, (2) epigenetic molecular memory, (3) allostatic load and systemic physiological adaptation, (4) the Psychological–Neurological–Endocrine–Immunological (PNEI) network, and (5) interoceptive–neuronal integration. By treating dementias as emergent outcomes of circular, bidirectional interactions across these levels, the framework aims to (a) reconcile key clinical and epidemiological paradoxes, (b) generate testable predictions regarding biomarkers and multidomain interventions, and (c) provide a conceptual roadmap for a more integrative, systems-oriented dementia research agenda.

## 2 The hierarchical-circular model of biological memory: conceptual framework

### 2.1 The unifying principle: “Signal → Plasticity → Stable State”

At the core of the Hierarchical-Circular Model lies a unifying principle applicable across all levels of biological organization: Signal → Plasticity → Stable State. This principle posits that biological systems continuously receive signals from their internal and external environments, undergo plastic changes in response to these signals, and subsequently settle into stable states that encode the history of prior signals. This triadic dynamic operates iteratively, such that each stable state becomes the basis for responding to subsequent signals, establishing circular causality across time.

This conceptualization draws from foundational work in systems biology and developmental biology. McNamara et al. (2024) demonstrated how morphogen signals drive cellular plasticity and establish stable spatial patterns during gastruloid development, illustrating the “Signal → Plasticity → Stable State” dynamic at the level of tissue patterning. Similarly, in neuroscience, Barrett and Simmons (2015) proposed that the brain operates as a predictive organ that continuously generates interoceptive predictions (signals), updates internal models through plasticity, and settles into stable allostatic states that shape subsequent predictions. The model extends this logic across multiple hierarchical levels, from molecular to systemic.

Critically, this principle operates circularly: stable states feedback to modulate how subsequent signals are detected and processed. When this circular dynamic functions adaptively, the organism maintains allostatic integrity—the capacity to flexibly respond to challenges while preserving core functional stability. When disruptions occur at any level, maladaptive stable states can become entrenched, propagating dysfunction across the system and eventually manifesting as dementia.

Boundaries of the principle: The principle “Signal → Plasticity → Stable State” does NOT apply to: (a) monogenic disorders with fully penetrant causal mutations (e.g., autosomal dominant AD due to APP, PSEN1, PSEN2), where a single-level disruption is sufficient to produce disease; (b) acute cognitive impairment due to isolated structural brain lesions without systemic propagation (e.g., single territorial stroke); or (c) normal developmental milestones that do not involve maladaptive consolidation of pathological stable states. The model is explicitly proposed for sporadic, late-onset, multifactorial dementias.

### 2.2 Level 1: morphogenetic programming and genetic architecture

The first level encompasses the genetic blueprint that establishes baseline vulnerabilities and resilience capacities across the lifespan. This includes constitutive genetic variants—such as APOE ε2/ε3/ε4 alleles—that modulate lipid metabolism, neuroinflammation, and synaptic integrity (Verduzco Espinoza et al., 2025). However, genetic architecture is not merely deterministic; it operates through dynamic interactions with environmental signals, a principle encapsulated in the concept of differential susceptibility (Belsky and Pluess, 2009).

Morphogenetic programming refers to the processes by which genetic information guides tissue organization, neural circuit formation, and organ system development during critical windows. Early-life signals—including nutritional status, stress exposure, and maternal care—can calibrate these developmental trajectories, establishing set points that persist into adulthood (Danese and McEwen, 2012). Importantly, genetic variants influence the degree to which environmental signals induce plastic changes, creating gene–environment interactions that shape individual differences in dementia risk.

Within the model, Level 1 provides the foundational architecture upon which higher-level processes operate. However, consistent with circular dynamics, information from other levels, particularly epigenetic modifications (Level 2) and allostatic load (Level 3), can feed back to influence gene expression patterns, effectively updating the functional consequences of the genetic blueprint across the lifespan.

### 2.3 Level 2: epigenetic molecular memory

The second level captures the molecular mechanisms through which environmental signals become biologically inscribed and maintained over time. Epigenetic modifications—including DNA methylation, histone acetylation, and non-coding RNA regulation—constitute a form of molecular memory that encodes past experiences and shapes future cellular responses (Zannas et al., 2016; Yusupov et al., 2024).

Work on stress-responsive genes has been particularly illuminating. Klengel et al. (2013) demonstrated that childhood trauma induces allele-specific DNA demethylation of FKBP5, a key regulator of glucocorticoid receptor sensitivity, leading to long-term alterations in HPA axis reactivity. Nöthling et al. (2025) extended these findings by showing that FKBP5 methylation patterns mediate the relationship between childhood trauma and stress-related psychopathology. Similarly, Panitch et al. (2024) identified APOE genotype-specific methylation patterns linked to Alzheimer’s disease pathology and estrogen response, illustrating how genetic architecture (Level 1) and epigenetic memory (Level 2) interact to shape disease risk.

Crucially, epigenetic marks are not static; they can be modified by ongoing experiences across the lifespan, including psychosocial stressors, metabolic states, and inflammatory signals (Feng et al., 2025). Within the circular framework, epigenetic memory serves as a bridge between genetic predisposition and systemic physiological adaptation, translating environmental signals into lasting biological changes that influence vulnerability to dementia.

### 2.4 Level 3: allostatic load and systemic physiological adaptation

The third level encompasses the cumulative physiological consequences of repeated or chronic adaptation to environmental demands. The concept of allostatic load, introduced by McEwen and Stellar (1993), operationalizes the “wear and tear” on the body resulting from sustained activation of stress-response systems, including the HPA axis, autonomic nervous system, and inflammatory pathways.

Allostatic load is typically measured through composite indices that integrate markers from multiple physiological systems—cardiovascular (e.g., blood pressure, heart rate variability), metabolic (e.g., body mass index, glycated hemoglobin), and inflammatory (e.g., C-reactive protein, interleukin-6) (Guidi et al., 2021). Longitudinal studies have demonstrated that elevated allostatic load is prospectively associated with all-cause dementia and Alzheimer’s disease specifically (Ruan et al., 2025; Souza-Talarico et al., 2025). Importantly, allostatic load captures cumulative risk that may not be evident from any single biomarker alone, reflecting the integrated state of multiple physiological systems.

Within the model, Level 3 functions as a critical integrator of signals from higher (Levels 4–5) and lower (Levels 1–2) levels. Chronic psychological stress (Level 4) is associated with allostatic load accumulation, while epigenetic modifications (Level 2) influence how effectively individuals regulate stress responses. Conversely, elevated allostatic load may feedback to induce further epigenetic changes and exacerbate neuroinflammatory processes, establishing vicious cycles that may accelerate cognitive decline.

### 2.5 Level 4: the psychological–neurological–endocrine–immunological (PNEI) network

The fourth level captures the dynamic interactions among psychological processes, neurological function, endocrine signaling, and immune activity. This PNEI network embodies the bidirectional communication pathways that link mental states, brain function, peripheral physiology, and inflammatory responses (Dantzer et al., 2008; Slavich and Irwin, 2014).

Psychological stress activates the HPA axis and sympathetic nervous system, triggering release of cortisol and catecholamines that modulate immune cell trafficking and cytokine production (Feng et al., 2025). Chronic stress promotes a pro-inflammatory phenotype characterized by elevated levels of interleukin-6, tumor necrosis factor-alpha, and C-reactive protein, which can compromise the blood–brain barrier and promote neuroinflammation (Dantzer et al., 2008; Tyagi and Bartley, 2025). Conversely, peripheral inflammation signals the brain through multiple pathways—including vagal afferents, circulating cytokines, and endothelial activation—inducing sickness behaviors, cognitive deficits, and neuroinflammation.

Within dementia pathogenesis, PNEI dynamics are increasingly recognized as central rather than peripheral. Li et al. (2025) demonstrated that inflammation-mediated regional brain alterations are associated with mild cognitive impairment in knee osteoarthritis, illustrating how peripheral inflammatory conditions can influence central pathology. Similarly, Verduzco Espinoza et al. (2025) showed that microglia-to-neuron signaling links APOE4 and inflammation to enhanced neuronal lipid metabolism and network activity, providing a mechanistic bridge between genetic risk, neuroimmune dynamics, and neural circuit function.

The PNEI network functions as a hub within the circular model, integrating signals from psychological states (e.g., stress, mood), neurological activity (e.g., network oscillations), endocrine status (e.g., cortisol rhythms), and immune function (e.g., cytokine profiles). Perturbations at any point in this network may propagate bidirectionally, potentially amplifying maladaptive states that increase dementia vulnerability.

### 2.6 Level 5: interoceptive–neuronal integration

The fifth level concerns the integration of interoceptive signals—sensory information about the internal state of the body—with neuronal dynamics across large-scale brain networks. Interoception, defined as the sense of the physiological condition of the body (Craig, 2002), provides the foundation for emotional experience, self-awareness, and adaptive regulation of internal states (Barrett and Simmons, 2015; Khalsa et al., 2018).

Recent advances have revealed that interoceptive processing relies on intrinsic neural timescales—the temporal windows over which neurons and neural populations integrate information. Hazelton et al. (2025a, b) demonstrated that patients with behavioral-variant frontotemporal dementia exhibit altered intrinsic neural timescales of interoceptive processing, with disruptions in allostatic–interoceptive networks that support adaptive regulation. Critically, these alterations were associated with clinical severity and predicted disease progression, suggesting that interoceptive dysfunction is not merely a correlate but potentially a driver of neurodegeneration.

Hazelton et al. (2025a, b) further showed, through neuroimaging meta-analyses, that interoception, emotion, and social cognition converge across neurodegenerative diseases, including Alzheimer’s disease. This convergence suggests that disturbances in interoceptive–neuronal integration may constitute a transdiagnostic mechanism of cognitive and behavioral decline. Within the model, interoceptive–neuronal integration represents the highest-order integrative level within the circular flow of information, where signals from genetic, epigenetic, physiological, and PNEI levels converge to shape conscious experience, self-regulation, and adaptive behavior.

**Figure 1:**
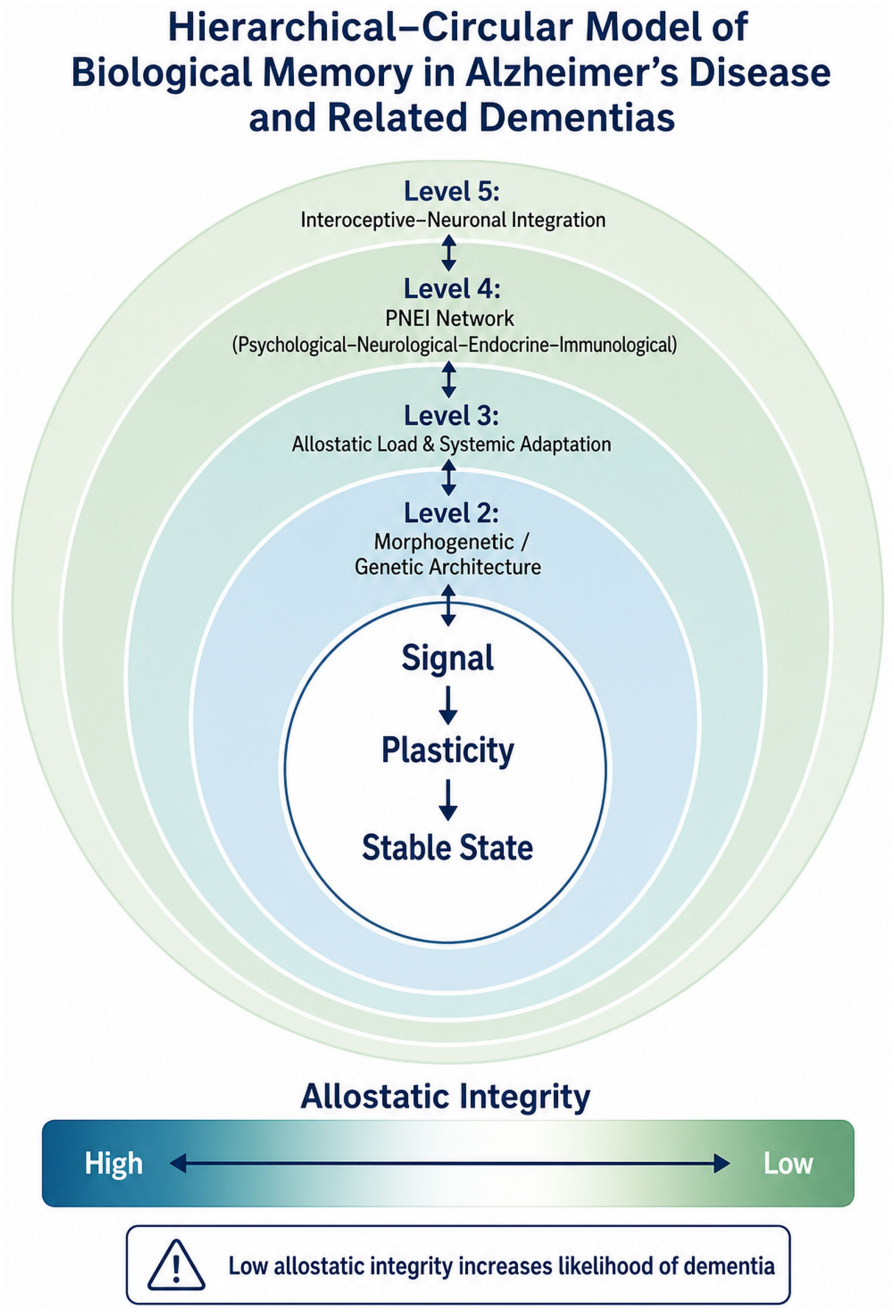
Hierarchical–circular model of biological memory in Alzheimer’s disease and related dementias. The model depicts five interconnected levels of biological memory—(1) morphogenetic/genetic architecture, (2) epigenetic molecular memory, (3) allostatic load and systemic adaptation, (4) the Psycho–Neuro–Endocrine–Immunological (PNEI) network, and (5) interoceptive–neuronal integration—organized around the unifying principle “Signal → Plasticity → Stable State.” Bidirectional arrows indicate circular information flow, whereby perturbations at any level can propagate through the system and consolidate maladaptive pathological stable states. Interoceptive–neuronal integration represents the highest-order integrative level within this circular flow. Low allostatic integrity increases the likelihood of progression to dementia.

When interoceptive–neuronal integration breaks down, the organism loses its capacity to accurately sense, predict, and regulate internal states, leading to maladaptive stable states that manifest clinically as cognitive impairment, behavioral dysregulation, and ultimately dementia (Figure 1).

### 2.7 Circular dynamics and bidirectional propagation across levels

A defining feature of the Hierarchical-Circular Model is the emphasis on bidirectional propagation of signals across levels. Unlike linear models that posit unidirectional causality (e.g., amyloid accumulation → neurodegeneration → cognitive decline), the circular model recognizes that perturbations at any level can propagate upward, downward, and laterally through the system.

For example:

- A genetic variant (Level 1) may influence epigenetic susceptibility (Level 2), which is associated with allostatic load accumulation (Level 3), affecting PNEI dynamics (Level 4) and interoceptive processing (Level 5). This represents upward propagation.
- Conversely, chronic psychological stress (Level 4) is associated with allostatic load (Level 3), which may correlate with epigenetic modifications (Level 2) that alter gene expression (Level 1). This represents downward propagation.
- Neuroimmune activation (Level 4) may directly influence interoceptive network function (Level 5) while simultaneously modulating allostatic load (Level 3), representing lateral propagation.

This circular architecture may explain why individuals with similar neuropathological burdens (e.g., amyloid and tau positivity) may follow markedly different clinical trajectories. When the system maintains allostatic integrity—the capacity to flexibly adapt to challenges while preserving circular information flow—neuropathology may be tolerated without clinical expression. When allostatic integrity breaks down, even modest pathology could trigger cascading dysfunction across levels, culminating in dementia.

The concept of allostatic integrity thus represents a dynamic systems-level property that cannot be reduced to any single biomarker or pathway. It is precisely this property that the Hierarchical-Circular Model seeks to operationalize and investigate.

### 2.8 Model boundaries and falsification criteria

What the model does NOT explain:

- Rare monogenic forms of dementia with fully penetrant causal mutations (e.g., APP, PSEN1, PSEN2)
- Acute cognitive impairment due to isolated structural brain lesions (e.g., single hemispheric stroke)
- Normal age-related cognitive changes without evidence of pathological circular reinforcement

Falsification criteria: The Hierarchical-Circular Model would be falsified if:

- Composite indices of allostatic integrity show no incremental predictive value for conversion from mild cognitive impairment to dementia beyond amyloid and tau status alone, in adequately powered longitudinal cohorts;
- Multidomain interventions targeting multiple levels show only additive effects (i.e., no statistical interaction, ANOVA interaction term *p* > 0.05) in properly designed randomized controlled trials;
- Individuals with preserved allostatic integrity universally progress to dementia regardless of their systemic physiological state, contradicting the moderation hypothesis.

These criteria ensure the model is empirically testable and not merely descriptive.

### 2.9 Relationship to reserve and resilience frameworks

The Hierarchical-Circular Model does not replace but rather integrates and extends the established constructs of cognitive reserve, brain reserve, and brain maintenance (Stern et al., 2020; Stern et al., 2023) (Table 1).

**Table 1:** Comparison of the construct of allostatic integrity with established reserve and resilience frameworks (cognitive reserve, brain reserve, and brain maintenance).

Construct	Definition	How allostatic integrity differs/extends
Cognitive reserve	Ability to maintain cognitive function despite pathology through flexible neural network use	Allostatic integrity is multisystem (not just neural); includes peripheral physiology, epigenetics, and interoception
Brain reserve	Structural brain features (e.g., head circumference, synapse density) that buffer against pathology	Allostatic integrity is dynamic (not static); changes across lifespan via circular feedback
Brain maintenance	Absence of pathological change despite risk factors	Allostatic integrity explicitly models positive adaptation (not just absence of pathology)

Integration: The model proposes that allostatic integrity modulates reserve expression. High allostatic integrity may enhance cognitive reserve through preserved interoceptive-prefrontal connectivity; low allostatic integrity may undermine reserve even when structural brain reserve is high. This integration generates discriminative predictions (see Section 4.4).

## 3 Development of the hypothesis: synthesis of evidence

Having established the conceptual architecture of the Hierarchical-Circular Model, this section synthesizes the empirical evidence that supports each level and the circular dynamics linking them. The evidence is drawn from longitudinal cohort studies, mechanistic investigations, and multisystem approaches spanning the 2010–2025 period, organized thematically around the model’s core components.

### 3.1 Gene–environment interactions and lifespan vulnerability

The foundation of the model rests on the premise that genetic architecture does not operate in isolation but interacts dynamically with environmental exposures across the lifespan. This gene–environment interplay is perhaps best exemplified by research on APOE and stress-related genes.

The APOE ε4 allele remains the strongest genetic risk factor for sporadic Alzheimer’s disease, yet its penetrance is far from complete. Verduzco Espinoza et al. (2025) demonstrated that APOE4 influences neuronal lipid metabolism and network activity through microglia-to-neuron signaling, revealing a mechanistic pathway that is modulated by inflammatory states. This finding suggests that the impact of APOE4 may depend on the inflammatory environment—an environmental factor that is itself shaped by stress exposure, diet, and other lifestyle variables.

Similarly, research on FKBP5 has elucidated how genetic variants and environmental stressors interact to calibrate stress reactivity. Zannas et al. (2016) showed that FKBP5 variants influence epigenetic regulation of the glucocorticoid receptor, with effects that are potentiated by early-life trauma. Klengel et al. (2013) demonstrated that childhood trauma induces allele-specific DNA demethylation of FKBP5, leading to long-term alterations in HPA axis reactivity. Nöthling et al. (2025) extended these findings by showing that FKBP5 methylation patterns mediate the relationship between childhood trauma and stress-related psychopathology in adulthood.

Longitudinal cohort studies have further established that early-life experiences are associated with dementia risk decades later. Livingston et al. (2020) and Fratiglioni et al. (2020) synthesized evidence showing that adverse childhood experiences, educational attainment, and socioeconomic status across the life course collectively contribute to dementia vulnerability. Lee et al. (2025) provided population-based evidence linking adverse experiences throughout the life course to increased dementia risk, reinforcing the notion that vulnerability is not determined at birth but emerges from cumulative gene–environment interactions.

Within the circular model, these findings illustrate upward propagation: genetic architecture (Level 1) shapes how environmental signals (e.g., childhood trauma) become biologically embedded through epigenetic mechanisms (Level 2), which in turn are associated with systemic physiology (Level 3) and psychological function (Level 4). Conversely, the model also predicts downward propagation: chronic stress (Level 4) may induce epigenetic modifications (Level 2) that alter gene expression patterns (Level 1), creating bidirectional associations.

#### 3.1.1 Illustrative mechanistic pathways of circular propagation

To move beyond abstract circularity, the model specifies concrete, testable mechanisms:

Pathway A: Peripheral inflammation → epigenetic modification → HPA dysregulation (bidirectional). Peripheral IL-6 crosses the blood–brain barrier → activates NF-κB in microglia → induces DNMT1-mediated hypermethylation of the NR3C1 (glucocorticoid receptor) promoter → reduces cortisol sensitivity → increases allostatic load → perpetuates inflammation. This specific pathway has been demonstrated in human cohorts (Tyrka et al., 2016; Davies et al., 2019).
Pathway B: Interoceptive dysfunction → chronic stress → epigenetic embedding (Levels 5 → 4 → 2). Altered anterior insula prediction signals → misinterpretation of bodily states → sustained sympathetic activation → elevated cortisol → induces FKBP5 demethylation at intron 7 → long-term HPA axis dysregulation. This pathway is supported by Hazelton et al. (2025a, b) and Klengel et al. (2013).
Pathway C: Allostatic load → neuroinflammation → interoceptive network disruption (Levels 3 → 4 → 5). Elevated allostatic load promotes peripheral cytokine production → activates brain endothelial cells → upregulates adhesion molecules → facilitates monocyte trafficking into the salience network (including anterior insula and anterior cingulate) → impairs interoceptive prediction accuracy → further increases allostatic load through poor self-regulation.

These pathways provide empirically testable causal links rather than abstract circularity.

### 3.2 Epigenetic embedding of stress and dementia risk

The second level of the model posits that epigenetic modifications constitute a form of molecular memory that encodes environmental exposures and shapes long-term vulnerability. Converging evidence from human and animal studies supports this proposition.

Yusupov et al. (2024) investigated DNA methylation patterns of FKBP5 regulatory regions in both brain and blood of humanized mice and humans, demonstrating concordance between central and peripheral tissues. This finding is critical because it suggests that peripheral epigenetic markers may serve as accessible proxies for brain epigenetic states, enabling large-scale human studies of epigenetic contributions to dementia risk.

Panitch et al. (2024) identified APOE genotype-specific methylation patterns linked to Alzheimer’s disease pathology and estrogen response, illustrating how epigenetic marks interact with genetic architecture. Their findings revealed that methylation at specific CpG sites modified the relationship between APOE genotype and neuropathological outcomes, suggesting that epigenetic variation may explain some of the heterogeneity in clinical expression among APOE ε4 carriers.

The concept of epigenetic embedding extends beyond single genes to encompass broader chromatin remodeling and non-coding RNA regulation. Feng et al. (2025) reviewed evidence showing that psychological stress induces widespread epigenetic changes that affect neuroinflammatory pathways, HPA axis function, and neural plasticity. These changes can persist for decades, effectively “programming” biological systems for either resilience or vulnerability.

Within the circular model, epigenetic memory serves as a bridge between genetic predisposition (Level 1) and systemic physiological adaptation (Level 3). Critically, epigenetic marks are not static; they can be modified by ongoing experiences, including psychosocial interventions, metabolic changes, and pharmacological agents (Feng et al., 2025). This plasticity opens possibilities for interventions that could reverse or mitigate maladaptive epigenetic states.

### 3.3 Allostatic load as a multisystem risk integrator

The third level of the model identifies allostatic load as a critical integrator of multisystem risk. Allostatic load operationalizes the cumulative physiological consequences of chronic stress across multiple regulatory systems, providing a composite measure that may outperform single biomarkers in predicting health outcomes.

Souza-Talarico et al. (2025) examined the relationship between allostatic load dynamics, Alzheimer’s disease biomarkers, and progression in individuals with mild cognitive impairment using data from the Alzheimer’s disease Neuroimaging Initiative. Their findings revealed that allostatic load trajectories were associated with conversion from mild cognitive impairment to dementia independently of amyloid and tau status, supporting the hypothesis that systemic physiological state modulates clinical expression of neuropathology.

Ruan et al. (2025) conducted a prospective cohort study demonstrating that elevated allostatic load was associated with increased risk for all-cause dementia and specific subtypes, including Alzheimer’s disease and vascular dementia. Pérez-Carrasco et al. (2025) extended these findings to a Chilean older-adults cohort, revealing distinct physiological aging trajectories that were associated with cognitive outcomes, suggesting that allostatic load captures culturally and contextually relevant aspects of physiological aging.

Smith et al. (2025) showed that allostatic load, as a measure of cumulative physiological stress, is associated with impaired cognitive resilience in older adults. Importantly, they demonstrated that allostatic load effects were independent of amyloid burden, suggesting that systemic physiology may contribute to cognitive decline through pathways distinct from classical neuropathological cascades.

The concept of allostatic load is grounded in foundational work by McEwen and Stellar (1993), who introduced the framework of allostasis—the process of achieving stability through physiological change. Guidi et al. (2021) provided a systematic review of allostatic load and its health impacts, confirming that composite indices consistently predict morbidity and mortality across diverse populations.

Within the circular model, allostatic load functions as an integrator of signals from multiple levels. Elevated allostatic load (Level 3) reflects cumulative input from genetic predisposition (Level 1), epigenetic regulation (Level 2), psychological stress (Level 4), and interoceptive dysfunction (Level 5). Conversely, elevated allostatic load may feedback to induce further epigenetic changes (Level 2) and exacerbate neuroinflammation (Level 4), establishing vicious cycles that may accelerate decline.

### 3.4 Neuroimmune dynamics and central–peripheral crosstalk

The fourth level of the model emphasizes the bidirectional communication between the central nervous system and peripheral immune processes. This neuroimmune axis has emerged as a critical determinant of brain health and dementia risk.

Dantzer et al. (2008) established the foundational framework for understanding how peripheral inflammation signals the brain, inducing sickness behaviors and cognitive deficits. Their work demonstrated that pro-inflammatory cytokines communicate with the brain through multiple pathways—including vagal afferents, circulating cytokine transport, and endothelial activation—triggering neuroinflammation that can persist long after the peripheral insult resolves.

Slavich and Irwin (2014) advanced this framework through the Social Signal Transduction Theory of Depression, which delineates how psychosocial stressors activate inflammatory responses that contribute to neuropsychiatric disorders. Their model provides a mechanistic bridge between psychological states (Level 4), immune function (Level 4), and brain health.

Feng et al. (2025) synthesized recent evidence on central–peripheral neuroimmune dynamics in psychological stress and depression, revealing bidirectional pathways through which stress alters immune function and immune signals modulate neural activity. They highlighted the role of the blood–brain barrier as a dynamic interface that regulates neuroimmune communication, with implications for dementia pathogenesis.

Tyagi and Bartley (2025) conducted a systematic review of dynamic cytokine relationships across the blood–brain barrier in humans and nonhuman primates, revealing complex patterns of central–peripheral coupling that vary across inflammatory states. Their findings suggest that the relationship between peripheral and central inflammation is not static but may depend on factors including chronicity, intensity, and individual genetic background.

Li et al. (2025) provided clinical evidence linking peripheral inflammation to brain alterations, demonstrating that inflammation-mediated regional brain changes were associated with mild cognitive impairment in knee osteoarthritis. This study illustrates how peripheral inflammatory conditions—in this case, osteoarthritis—can influence central pathology and cognitive function, supporting the model’s emphasis on central–peripheral crosstalk.

#### 3.4.1 Peripheral–central circular dynamics: evidence from systemic inflammation

Recent reviews highlight that peripheral inflammatory insults—including infections, metabolic syndrome, and gut dysbiosis—may contribute to Alzheimer’s pathogenesis through bidirectional brain–body signaling (Chen et al., 2025; Liu et al., 2023). These studies demonstrate that: (a) peripheral inflammation can precede central pathology by years; (b) systemic insults may amplify neuroinflammation via blood–brain barrier disruption and vagal afferent pathways; and (c) central inflammation may feedback to exacerbate peripheral immune dysregulation. This evidence directly supports the circular model’s claim that perturbations at Level 4 (PNEI) can propagate across levels: peripheral inflammation → neuroinflammation → allostatic load → epigenetic modification → further peripheral dysregulation.

Within the circular model, the PNEI network (Level 4) functions as a hub integrating signals from psychological, neurological, endocrine, and immune domains. Perturbations at any point may propagate through the network, with neuroimmune activation feeding back to allostatic load (Level 3) and interoceptive processing (Level 5).

### 3.5 Interoceptive dysfunction as a transdiagnostic mechanism

The fifth level of the model concerns interoceptive–neuronal integration, positing that disruptions in the brain’s capacity to sense, predict, and regulate bodily states may constitute a transdiagnostic mechanism of cognitive and behavioral decline.

Barrett and Simmons (2015) proposed a predictive coding framework for interoception, arguing that the brain continuously generates predictions about bodily states, updates these predictions through interoceptive afferents, and uses prediction errors to refine internal models. This framework positions interoception as fundamental to emotion, self-awareness, and adaptive regulation.

Khalsa et al. (2018) provided a comprehensive roadmap for interoception research in mental health, outlining measurement approaches, neural substrates, and clinical applications. Their review highlighted the relevance of interoception to conditions ranging from anxiety and depression to neurodegenerative diseases, supporting the transdiagnostic perspective.

Critchley and Garfinkel (2017) emphasized the role of interoceptive processing in allostatic regulation, linking interoceptive accuracy to emotion regulation, decision-making, and social cognition. Their work provides a foundation for understanding how interoceptive dysfunction may contribute to the behavioral and cognitive features of dementia.

Hazelton et al. (2025a, b) provided direct evidence linking interoceptive dysfunction to neurodegeneration in behavioral-variant frontotemporal dementia. Using advanced neuroimaging techniques, they demonstrated altered intrinsic neural timescales of interoceptive processing, with disruptions in allostatic–interoceptive networks that support adaptive regulation. Critically, these alterations were associated with clinical severity and predicted disease progression, suggesting that interoceptive dysfunction is not merely a correlate but potentially a driver of neurodegeneration.

Hazelton et al. (2025a, b) extended these findings through neuroimaging meta-analyses revealing convergence of interoception, emotion, and social cognition across neurodegenerative diseases, including Alzheimer’s disease. Their results suggest that disturbances in interoceptive–neuronal integration may constitute a common mechanism underlying diverse neurodegenerative conditions, supporting the model’s claim that interoceptive dysfunction is transdiagnostic.

Within the circular model, interoceptive–neuronal integration (Level 5) represents the highest-order integrative level within the circular flow of information. Signals from genetic (Level 1), epigenetic (Level 2), allostatic (Level 3), and PNEI (Level 4) levels converge in interoceptive networks that shape conscious experience, self-regulation, and adaptive behavior. When these networks are disrupted, the organism loses its capacity to accurately sense and regulate internal states, leading to maladaptive stable states that manifest clinically as dementia.

### 3.6 Synthesis: toward a unifying framework

Taken together, the evidence synthesized across Levels 1–5 reveals a convergent picture: dementia risk and progression are shaped by interactions among genetic architecture, epigenetic molecular memory, cumulative allostatic load, neuroimmune dynamics, and interoceptive–neuronal integration. Each of these domains has been investigated independently, but the circular model provides a framework for understanding how they may interact bidirectionally across the lifespan.

The concept of allostatic integrity—the capacity of the organism to maintain adaptive circular information flow across multiple levels of organization—emerges as a dynamic systems-level property that may explain why individuals with similar neuropathological burdens follow divergent clinical trajectories. When allostatic integrity is preserved, even substantial pathology may be tolerated without clinical expression. When allostatic integrity is compromised, even modest pathology could trigger cascading dysfunction across levels, culminating in dementia.

This synthesis supports the model’s central hypothesis: dementias are best understood not as isolated brain pathologies but as disorders of biological memory and allostatic integrity. The Hierarchical-Circular Model provides a conceptual roadmap for investigating this hypothesis through testable predictions, which are developed in the following section.

## 4 Testable predictions and empirical consequences

A central requirement of any scientific hypothesis is that it generates concrete, falsifiable predictions that can be tested through empirical investigation. The Hierarchical-Circular Model yields four core predictions that differentiate it from linear, single-pathway models of dementia pathogenesis. Each prediction is grounded in the model’s conceptual architecture and can be evaluated using existing longitudinal cohorts, biomarker panels, and intervention trial designs.

### 4.1 Prediction 1: composite indices of allostatic integrity will outperform single biomarkers in predicting conversion from mild cognitive impairment to dementia

If dementia emerges from disruptions in circular information flow across multiple levels of biological organization, then a composite measure capturing this integrated state should predict clinical outcomes more accurately than any single biomarker alone. This prediction directly challenges the prevailing assumption that a single molecular entity—such as amyloid-beta or tau—can serve as a sufficient predictive marker for clinical progression.

#### 4.1.1 What makes this prediction unique to the circular model

The model predicts that dyssynchrony profiles (e.g., high inflammation with low cortisol, indicating feedback loop failure) will have the strongest predictive power—not merely the sum of individual markers. Linear composites (e.g., additive allostatic load indices) are insufficient; dynamic synchrony across levels is what captures circular flow.

#### 4.1.2 Empirical support

Preliminary evidence is consistent with this prediction. Souza-Talarico et al. (2025) demonstrated that allostatic load trajectories were associated with conversion from mild cognitive impairment to dementia independently of amyloid and tau status. Ruan et al. (2025) and Smith et al. (2025) similarly showed that allostatic load was associated with dementia risk beyond traditional biomarkers. However, these studies used allostatic load indices that primarily capture Level 3 (systemic physiological adaptation). The model predicts that composite indices incorporating markers from Levels 2 (epigenetic), 4 (neuroimmune), and 5 (interoceptive) will demonstrate even stronger predictive validity.

#### 4.1.3 Testable approach

Using existing longitudinal cohorts such as the Alzheimer’s Disease Neuroimaging Initiative (ADNI), the Religious Orders Study (ROS), or the Rush Memory and Aging Project (MAP), researchers can construct composite indices of allostatic integrity that integrate: Level 2 (DNA methylation patterns at stress-related loci such as FKBP5, NR3C1), Level 3 (multi-system allostatic load indices), Level 4 (inflammatory cytokine profiles, HPA axis measures, and autonomic function), and Level 5 (interoceptive accuracy measures and functional connectivity within salience and interoceptive networks). The model predicts that such composite indices will show higher area under the curve (AUC) values in predicting conversion from mild cognitive impairment to dementia compared to amyloid PET status, CSF p-tau181, or any single biomarker alone. Moreover, the model predicts that allostatic integrity indices will be associated with conversion even among individuals who are amyloid-positive, explaining variance in clinical trajectories that current frameworks cannot account for.

### 4.2 Prediction 2: multidomain interventions targeting more than one system level will have multiplicative, rather than additive, effects on slowing cognitive decline

If the system is characterized by circular, bidirectional interactions across levels, then interventions that target multiple levels simultaneously should produce synergistic—not merely additive—effects. This prediction has profound implications for clinical trial design and prevention strategies.

#### 4.2.1 What makes this prediction unique

While multidomain interventions exist (e.g., FINGER trial), the circular model uniquely predicts multiplicative effects (statistical interaction, not just additivity). This is falsifiable: if the effect of combined intervention equals the sum of single interventions (ANOVA interaction term *p* > 0.05), the model is contradicted. Current literature shows additive effects; the model predicts synergy.

#### 4.2.2 Empirical support

The FINGER trial (Kivipelto et al., 2017) provided early evidence that multidomain interventions (diet, exercise, cognitive training, vascular risk management) can slow cognitive decline in at-risk older adults. Subsequent trials have extended this approach, though few have systematically tested multiplicative versus additive effects. The model predicts that the magnitude of benefit from multidomain interventions reflects not merely the sum of individual component effects but their synergistic interactions across levels.

#### 4.2.3 Testable approach

Randomized controlled trials can be designed to test this prediction by comparing: Group A (single-domain intervention, e.g., anti-amyloid therapy alone); Group B (single-domain intervention targeting a different level, e.g., psychosocial stress reduction alone); Group C (combined intervention targeting both levels); and Group D (combined intervention targeting three or more levels, e.g., anti-amyloid + stress reduction + metabolic optimization + cognitive training). The model predicts that Group C will show effects greater than the sum of Groups A and B (multiplicative interaction), and Group D will show progressively larger multiplicative effects. These effects can be quantified using interaction terms in linear mixed models or through causal mediation analysis that examines whether combined interventions disrupt circular dynamics more effectively than single interventions. The model predicts that the multiplicative effects will be most pronounced among individuals with high allostatic load or compromised allostatic integrity at baseline, as these individuals may be most vulnerable to circular reinforcement of maladaptive states.

### 4.3 Prediction 3: patients with similar amyloid/tau profiles but contrasting allostatic integrity will show markedly different trajectories of clinical progression

Perhaps the most clinically significant prediction of the model is that allostatic integrity—not neuropathological burden alone—may determine clinical trajectory. This prediction directly addresses the long-standing paradox of dissociation between pathology and clinical expression.

#### 4.3.1 What makes this prediction unique

The model uniquely predicts that allostatic integrity will moderate the relationship between neuropathological burden and cognitive decline. In linear models, pathology and outcome are assumed monotonic. The circular model predicts a statistical crossover interaction: high allostatic integrity flattens the pathology–decline slope; low allostatic integrity steepens it. This pattern is not predicted by single-pathway models.

#### 4.3.2 Empirical support

Savva et al. (2009) first systematically documented the dissociation between neuropathological burden and clinical dementia in the Medical Research Council Cognitive Function and Ageing Study. More recent work by Souza-Talarico et al. (2025) showed that allostatic load dynamics were associated with conversion independently of amyloid and tau, suggesting that systemic physiology may moderate the clinical impact of neuropathology. However, no study to date has directly tested whether allostatic integrity indices predict divergent trajectories among individuals matched for amyloid and tau status.

#### 4.3.3 Testable approach

Using longitudinal cohorts with both neuroimaging biomarkers and comprehensive multi-system data, researchers can identify subgroups of individuals who are: amyloid-positive/tau-positive with high allostatic integrity; amyloid-positive/tau-positive with low allostatic integrity; amyloid-negative/tau-negative with high allostatic integrity; and amyloid-negative/tau-negative with low allostatic integrity. The model predicts that among amyloid-positive/tau-positive individuals, those with high allostatic integrity will show slow or no cognitive decline over follow-up, whereas those with low allostatic integrity will show rapid progression to dementia. Conversely, among amyloid-negative/tau-negative individuals, those with low allostatic integrity may show cognitive decline despite absence of classical neuropathology, potentially representing non-Alzheimer’s dementia phenotypes. This prediction can be tested using existing cohorts such as ADNI, ROSMAP, or the Harvard Aging Brain Study. The critical innovation is stratifying by both neuropathological status and allostatic integrity, rather than examining either in isolation.

### 4.4 Prediction 4 (discriminative): allostatic integrity moderates reserve expression

To differentiate the model from reserve frameworks (Stern et al., 2020, 2023), the circular model generates a discriminative prediction:

Among individuals matched for neuropathological burden and cognitive reserve proxies (e.g., education, occupational complexity, intracranial volume), those with low allostatic integrity will show faster clinical progression than those with high allostatic integrity. Conversely, among individuals matched for allostatic integrity, the protective effect of cognitive reserve will be attenuated when allostatic integrity is low.

This crossover pattern is unique to the circular model. Reserve frameworks alone predict that high reserve protects regardless of peripheral state. The circular model predicts that allostatic integrity moderates reserve expression: systemic dysregulation (inflammation, HPA axis dysfunction, interoceptive impairment) can override neural-level reserve. This is testable by stratifying ADNI participants by both reserve proxies and allostatic integrity indices.

## 5 Discussion

The Hierarchical-Circular Model of Biological Memory proposes a fundamental reconceptualization of Alzheimer’s disease and related dementias. Rather than viewing dementias as linear consequences of aggregated proteins, the model frames them as emergent outcomes of disrupted circular information flow across five interconnected levels: genetic architecture, epigenetic molecular memory, allostatic load, PNEI network dynamics, and interoceptive–neuronal integration. This discussion addresses the model’s capacity to reconcile clinical paradoxes, its relationship to existing frameworks, its implications for research and clinical practice, its limitations, and directions for future investigations.

### 5.1 Relationship to reserve and resilience frameworks

Stern et al. (2020, 2023) provided the definitive framework for cognitive reserve, brain reserve, and brain maintenance in Alzheimer’s disease. The Hierarchical-Circular Model complements rather than competes with this framework. While reserve models explain inter-individual differences in the relationship between pathology and cognition, they do not specify how peripheral physiology, epigenetic embedding, or interoceptive function contribute to reserve expression. The present model proposes that allostatic integrity is a determinant of reserve expression: individuals with high allostatic integrity may more effectively deploy cognitive reserve strategies; those with low allostatic integrity may have their reserve capacity undermined by chronic inflammation, HPA dysregulation, or interoceptive dysfunction. This integration generates Prediction 4 (Section 4.4).

### 5.2 Operationalizing allostatic integrity: toward a composite index

A critical next step is developing validated composite indices of allostatic integrity. The model proposes the following provisional components (Table 2):

**Table 2:** Proposed components of an allostatic integrity index.

Domain	Proposed markers	Provisional weighting
Adaptive reserve	HRV (high frequency), cortisol awakening response (normal slope), DHEA/cortisol ratio	25%
Inflammatory resilience	IL-10/IL-6 ratio, CRP (<3 mg/L), TNF-α	20%
Multi-system synchrony	Correlation between epigenetic age (Horvath clock) and physiological stress markers	20%
Interoceptive accuracy	Heartbeat detection task accuracy, anterior insula functional connectivity	20%
Circular recovery	Speed of return to baseline (cortisol, HRV, cytokines) after acute stress challenge	15%

Distinction from related constructs:

- Allostatic load = cumulative wear and tear (higher values = worse)
- Allostatic integrity = dynamic adaptive capacity (higher values = better)
- Frailty index = deficit accumulation (broader, includes functional decline)
- Biological aging metrics = epigenetic clocks (one component, not integrative)

Provisional formula: Allostatic Integrity Score = *Σ* (wᵢ × zᵢ) where wᵢ are weights and zᵢ are standardized scores, with higher scores indicating greater adaptive capacity. This operationalization requires empirical validation in future studies.

### 5.3 Reconciling clinical paradoxes through a systems Lens

The model’s most immediate contribution is its ability to reconcile several long-standing paradoxes that linear models struggle to explain.

Paradox 1: Dissociation between pathology and clinical expression. The observation that individuals with substantial amyloid and tau pathology may remain cognitively intact while others with minimal pathology decline rapidly (Savva et al., 2009) has challenged the amyloid cascade hypothesis for decades. The circular model resolves this paradox by positing that neuropathology is one input among many into a dynamic system. When allostatic integrity is preserved, the system can tolerate substantial pathology without clinical expression. When allostatic integrity is compromised, even modest pathology could trigger cascading dysfunction across levels. Allostatic integrity thus may function as a moderator of pathology’s clinical impact.

Paradox 2: The role of early-life stress in late-life dementia. The finding that childhood adversity is associated with dementia risk decades later (Livingston et al., 2020; Lee et al., 2025) is difficult to reconcile with models that focus on late-life protein aggregation. The circular model explains this through epigenetic embedding: early-life stress induces lasting epigenetic modifications (Klengel et al., 2013; Yusupov et al., 2024) that calibrate stress reactivity and allostatic load dynamics across the lifespan. These early influences may propagate upward through the system, shaping vulnerability decades before clinical symptoms emerge.

Paradox 3: The failure of single-target therapies. Despite clear evidence that amyloid and tau are involved in Alzheimer’s disease, anti-amyloid therapies have produced only modest clinical benefits (Cummings et al., 2024). The circular model explains this by emphasizing that dementias are systems-level disorders; targeting a single molecular entity may not fully restore circular information flow when multiple levels are dysregulated. This does not imply that anti-amyloid therapies are without value, but rather that they may need to be combined with interventions targeting other levels to achieve meaningful clinical effects—a prediction consistent with the multiplicative effects hypothesis (Prediction 2).

### 5.4 Implications for biomarker development and longitudinal cohorts

The model has concrete implications for how biomarkers are developed and how longitudinal cohorts are designed.

From single biomarkers to composite indices. Current biomarker strategies in Alzheimer’s disease research have focused on identifying single molecular entities (amyloid, tau, neurodegeneration) that correlate with pathology. The model suggests that composite indices capturing allostatic integrity across multiple levels will have greater predictive validity. This implies a shift toward multi-modal biomarker panels that integrate genetic markers (Level 1), epigenetic markers (Level 2), multi-system physiological markers (Level 3), neuroimmune and endocrine markers (Level 4), and interoceptive and functional connectivity markers (Level 5).

Longitudinal cohort design. Existing cohorts such as ADNI, ROSMAP, and the UK Biobank have collected rich multi-domain data, enabling initial tests of the model’s predictions. However, future cohorts should be designed explicitly to capture circular dynamics across levels. This requires repeated measures across multiple time points to model dynamic trajectories, integration of central and peripheral measures to capture central–peripheral crosstalk, assessment of interoceptive function using validated behavioral and neuroimaging paradigms, and collection of early-life exposure data to model epigenetic embedding. The model also suggests that allostatic integrity should be treated as a primary outcome in its own right, rather than merely a predictor of cognitive decline. Interventions that restore allostatic integrity may confer resilience even without directly reducing neuropathological burden.

### 5.5 Implications for multidomain prevention and personalized treatment

The model’s emphasis on circular dynamics across multiple levels has profound implications for prevention and treatment strategies.

Multidomain prevention. The FINGER trial (Kivipelto et al., 2017) demonstrated that multidomain interventions can slow cognitive decline in at-risk older adults. The circular model provides a mechanistic rationale for this approach: because levels are coupled through bidirectional feedback loops, interventions targeting multiple levels may disrupt maladaptive circular dynamics more effectively than single-domain approaches. The model further predicts that such interventions will show multiplicative, rather than additive, effects (Prediction 2)—a prediction that can be tested in future trials.

Personalized treatment based on allostatic integrity profiles. If allostatic integrity moderates the clinical impact of neuropathology, then treatment strategies should be tailored based on an individual’s allostatic integrity profile. Individuals with high allostatic integrity may tolerate substantial pathology without requiring aggressive anti-amyloid therapy. Conversely, individuals with low allostatic integrity may benefit from interventions targeting the specific levels where their system is most dysregulated—whether that be epigenetic (Level 2), allostatic (Level 3), PNEI (Level 4), or interoceptive (Level 5)—in addition to any anti-pathology therapies. This personalized approach aligns with broader movements toward precision medicine, but with a crucial difference: rather than stratifying patients solely by genetic or molecular biomarkers, the model advocates stratification by systems-level integrity—a dynamic property that integrates information across multiple levels of biological organization.

### 5.6 Limitations and future directions

While the Hierarchical-Circular Model offers a unifying framework, several limitations must be acknowledged and addressed through future research.

Complexity and testability. The model’s strength—its integration of multiple levels—is also a source of complexity. Testing the model’s predictions requires multi-modal data, sophisticated analytical approaches (e.g., dynamical systems modeling, causal mediation analysis), and large sample sizes to detect interaction effects. However, the model is explicitly designed to generate falsifiable predictions, and existing cohorts provide initial opportunities for empirical testing.

Causality vs. Correlation. Much of the evidence synthesized in Section 3 is correlational. Establishing causality requires intervention studies that manipulate specific levels and observe effects across the system. The model predicts that interventions targeting multiple levels will produce multiplicative effects, providing a strong test of causal circular dynamics.

Generalizability across dementia subtypes. The model was developed with Alzheimer’s disease as the primary focus, but its principles may extend to other dementias. Hazelton et al. (2025a, b) showed convergence of interoceptive, emotional, and social cognitive disruptions across neurodegenerative diseases, suggesting that the model’s transdiagnostic claims are plausible. Future work should test whether allostatic integrity predicts outcomes in vascular dementia, frontotemporal dementia, and Lewy body dementia.

Operationalizing allostatic integrity. A critical next step is developing validated composite indices of allostatic integrity that can be used across studies. Section 5.2 provides a provisional operationalization, but empirical work is needed to refine these indices, determine optimal weights, and validate them across diverse populations.

## 6 Conclusion

The Hierarchical-Circular Model of Biological Memory offers a unifying hypothesis for Alzheimer’s disease and related dementias that bridges genetic, epigenetic, physiological, neuroimmune, and interoceptive processes across the lifespan. By reframing dementias as disorders of biological memory and allostatic integrity—rather than isolated brain pathologies—the model resolves key clinical paradoxes, generates testable predictions, and provides a conceptual roadmap for mechanistic research, multidomain prevention, and personalized treatment strategies.

The model’s central contribution is the concept of allostatic integrity: the capacity of the organism to maintain adaptive circular information flow across multiple levels of organization. When allostatic integrity is preserved, the system may tolerate substantial neuropathology without clinical expression. When allostatic integrity is compromised, even modest pathology could trigger cascading dysfunction across levels, culminating in dementia.

The four core predictions derived from the model—(1) composite indices of allostatic integrity will outperform single biomarkers in predicting conversion from mild cognitive impairment to dementia; (2) multidomain interventions targeting more than one system level will have multiplicative effects on slowing cognitive decline; (3) patients with similar amyloid/tau profiles but contrasting allostatic integrity will show divergent clinical trajectories; and (4) allostatic integrity moderates the protective effect of cognitive reserve—provide clear opportunities for empirical testing using existing cohorts and trial designs.

Ultimately, the Hierarchical-Circular Model invites a shift in perspective: from viewing dementias as diseases of the brain in isolation to understanding them as failures of the organism’s capacity to maintain integrated, adaptive information flow across the lifespan. This reframing opens new avenues for research and intervention, emphasizing resilience, systems-level integrity, and the dynamic interplay between genes, environment, physiology, and experience.

## Data Availability

The original contributions presented in the study are included in the article/supplementary material, further inquiries can be directed to the corresponding author.
